# The Atypical Presentation of Diffuse Large B-cell Lymphoma in a Lower Limb Soft Tissue Mass: A Case Report

**DOI:** 10.7759/cureus.86676

**Published:** 2025-06-24

**Authors:** Filipe Leal, Amélia Gaspar, Cristina Silva, Clarisse Coelho, Ana Sofia Martins

**Affiliations:** 1 Family Health Unit, USF Mondego, Coimbra Local Health Unit, Coimbra, PRT

**Keywords:** diagnosis delay, diffuse large b-cell lymphoma, extranodal involvement, geriatric, soft tissue masses

## Abstract

We report a case of an 80-year-old male with a progressively enlarging, painful mass in the left lower limb, initially misdiagnosed as cellulitis. The patient did not respond to antibiotic therapy, prompting further investigation. Imaging revealed two large soft tissue masses, and biopsy confirmed diffuse large B-cell lymphoma (DLBCL) of germinal center origin. Despite treatment with R-CHOP (rituximab, cyclophosphamide, doxorubicin, vincristine, and prednisone) chemotherapy and palliative radiotherapy, the disease progressed, and the patient died approximately one year after diagnosis. This report illustrates the diagnostic challenge posed by extranodal DLBCL when it mimics common soft tissue infections. Delayed diagnosis in our case was caused by the benign clinical presentation and limited access to imaging in the primary care setting. Clinicians should maintain a high index of suspicion for malignancy in atypical or unresponsive soft tissue lesions. Early referral, multidisciplinary evaluation, and appropriate imaging are critical to improving outcomes in such rare presentations.

## Introduction

Family doctors frequently encounter patients with vague and nonspecific symptoms, which are often attributed to common and benign conditions. However, in some cases, they may represent the initial or atypical presentation of serious diseases, including malignancies such as lymphomas. Diffuse large B-cell lymphoma (DLBCL) is the most common subtype of non-Hodgkin lymphoma, accounting for 30-40% of cases. It typically affects older adults and is characterized by rapid clinical progression [1,2]. While DLBCL often presents with painless lymphadenopathy or systemic B symptoms, up to 30% of patients may exhibit extranodal involvement, affecting sites such as the gastrointestinal tract, CNS, bone, and, rarely, soft tissues and skin [2,3].

Soft tissue and skin lymphomas may be difficult to distinguish from other conditions, particularly in the absence of systemic symptoms. They may mimic cellulitis, abscesses, or vascular disorders, which are far more common in primary care. Consequently, when a lesion fails to respond to standard treatment, further investigation, including imaging, biopsy, or referral to a different level of care, should be pursued [3,4,5]. We discuss a case of an unusual manifestation of DLBCL in an elderly male, initially presenting as a rapidly enlarging lower limb soft tissue mass misinterpreted as a bacterial skin infection. This report vividly illustrates the diagnostic challenges inherent in atypical presentations and underscores the critical role of vigilance, reassessment, and multidisciplinary coordination in primary care.

Written informed consent was obtained from the patient for the publication of clinical data.

## Case presentation

**Figure 1:**
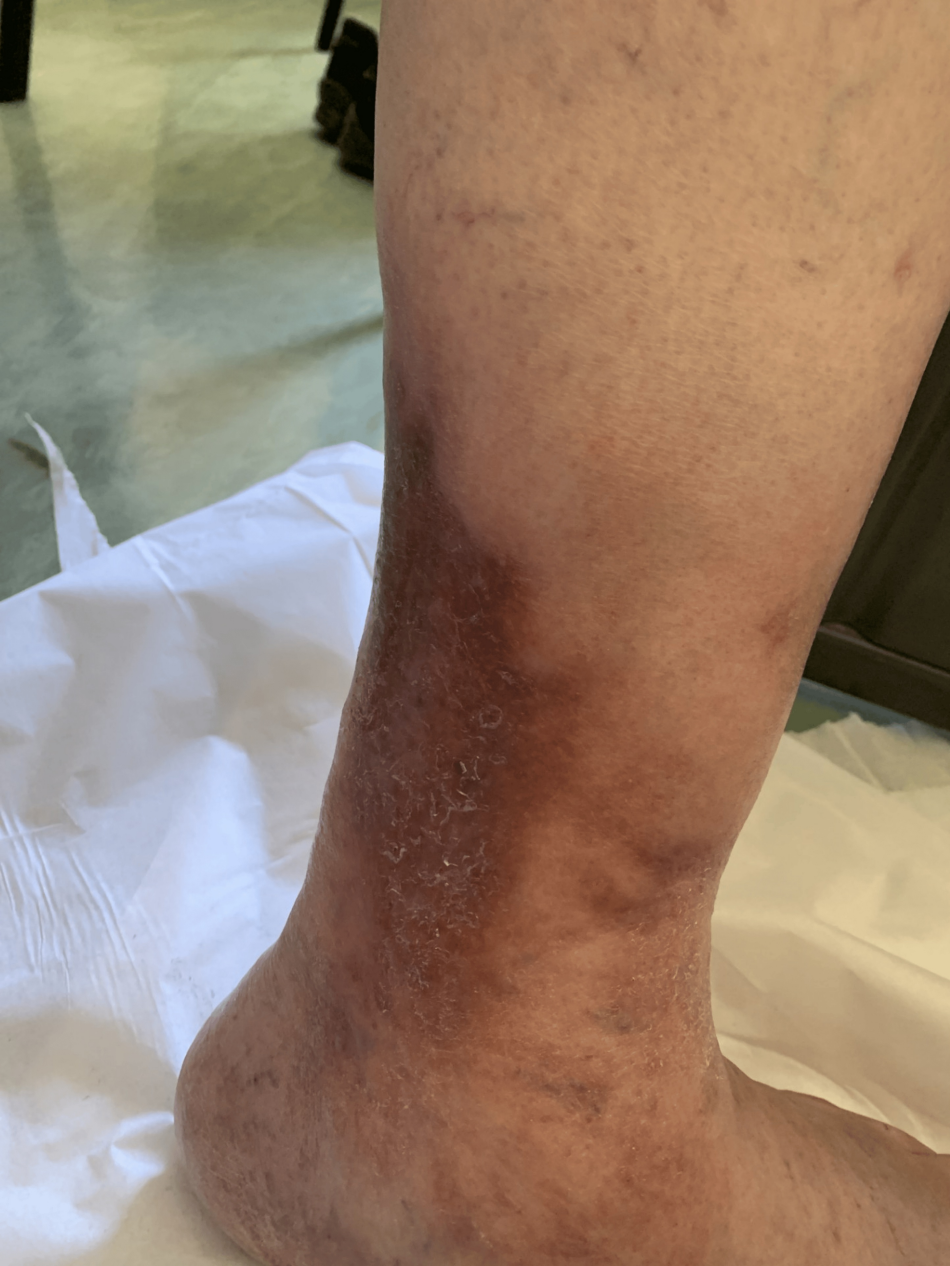
Clinical image showing swelling and redness affecting the lower third of the patient’s left leg at initial presentation

An 80-year-old male with a history of hypertension and no prior oncologic disease, in good general condition, presented to primary care with pain, swelling, and erythema in the left ankle and lower leg (Figure 1). The patient had no other associated symptoms, such as fever, weight loss, fatigue, night sweats, or palpable lymphadenopathy. The initial impression was bacterial cellulitis, and he was treated with flucloxacillin. As symptoms persisted, clindamycin was added, and a soft tissue ultrasound with Doppler was performed, revealing a “nodular formation adjacent to the popliteal nerve”, which, at the time, was not definitively characterized as malignant. MRI was suggested, but it was unavailable in primary care, and the patient was referred to Orthopedics for further evaluation.

**Figure 2:**
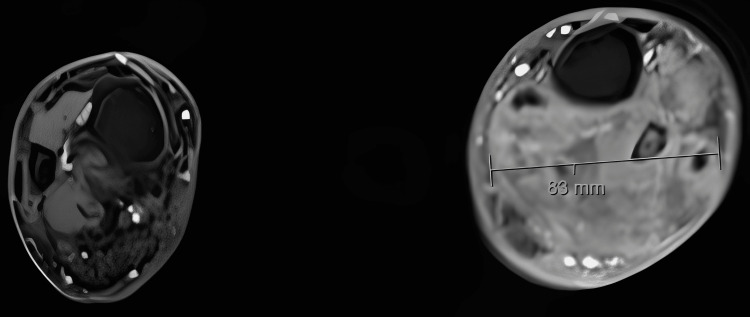
MRI revealing a soft tissue lesion involving the left fibula, measuring approximately 8 cm in transverse dimensions MRI: magnetic resonance imaging

One month later, the patient returned to primary care with worsening edema and significant limb enlargement. A CT scan revealed two well-defined soft tissue masses: one in the popliteal region (14 × 8 × 6.5 cm) and another in the distal leg (13 × 7 × 5 cm). Ultrasound-guided biopsy, performed approximately three months after initial presentation, confirmed DLBCL of germinal center origin, and the patient was referred to Hematology. A PET scan revealed widespread lymphadenopathy and two hypermetabolic soft tissue masses in the left leg, and MRI showed “Two solid soft tissue masses suggestive of metastatic nature located in the posterior compartment of the knee, associated with popliteal vein thrombosis, and in the left ankle involving the peroneal region” (Figure 2). The patient was started on R-CHOP (rituximab, cyclophosphamide, doxorubicin, vincristine, and prednisone) chemotherapy. Re-staging PET showed reduced metabolic activity but increased mass size. Given vascular involvement, surgical resection was not an option. He received palliative radiotherapy, which offered symptomatic relief. Despite second-line chemotherapy, the patient died one year after diagnosis.

## Discussion

DLBCL frequently presents with nodal enlargement and "B symptoms" such as fever, weight loss, and night sweats [1,2,5]. Extranodal presentations account for up to 40% of cases, involving sites such as the gastrointestinal tract, central nervous system, and bone marrow [5,6,7]. However, primary cutaneous or soft tissue involvement remains rare and can closely mimic infectious, inflammatory, or even vascular conditions [3,6,7,8]. This diagnostic ambiguity is particularly pronounced in elderly patients, in whom comorbidities such as chronic venous insufficiency can obscure early neoplastic signs [9,10]. Our patient's initial presentation involved a rapidly enlarging soft tissue mass in the lower limb, accompanied by localized inflammatory signs and underlying venous stasis. These features, in conjunction with the absence of systemic symptoms, led to a presumptive diagnosis of cellulitis - a far more prevalent and plausible condition in the elderly population and those with vascular disease. The initial clinical judgment was reasonable, reflecting common practice; however, the persistent symptoms and progressive nature of the lesion, despite empirical antibiotic therapy, prompted further investigation.

Imaging played a critical role in redirecting the diagnostic process. However, limited access to advanced imaging modalities such as MRI in Portuguese primary care settings likely contributed to a delay in definitive diagnosis. This report underscores a broader systemic issue regarding disparities in diagnostic resources, which may disproportionately affect older or rural patients. Given that delayed diagnosis adversely affects the prognosis of aggressive lymphomas such as DLBCL, the three-month interval between symptom onset and biopsy may have significantly contributed to the poor outcome observed.

Once diagnosed, DLBCL requires urgent and coordinated management. The standard first-line treatment remains the R-CHOP regimen, which has markedly improved survival rates [4,6,8]. Nonetheless, certain biological subtypes-particularly the activated B-cell-like (ABC) variant-are associated with poorer outcomes and higher relapse rates despite standard therapy [4,8,10]. In this patient, the initial response was suboptimal, with continued disease progression despite a reduction in metabolic activity on the PET scan. This highlights the aggressive and sometimes refractory nature of certain DLBCL subtypes, emphasizing the need for early consideration of second-line regimens such as salvage chemotherapy (e.g., R-ICE or R-DHAP), autologous stem cell transplantation, or novel agents, including CAR T-cell therapy in eligible patients.

This case also highlights the crucial role of multidisciplinary collaboration among primary care, orthopedics, and hematology. The family physician was instrumental in identifying the unusual course of what initially appeared to be a common infection, initiating the diagnostic workup, and ensuring timely referral, despite limitations in access to advanced diagnostic tools. This reinforces the need for heightened clinical vigilance and the value of reassessment in cases with atypical or non-resolving features. Finally, this case serves as a reminder that lymphoproliferative disorders should be included in the differential diagnosis of persistent or enlarging soft tissue lesions, particularly in older adults, even in the absence of classic systemic features or palpable lymphadenopathy. An open and dynamic diagnostic approach, incorporating timely imaging and biopsy, is crucial to avoid misdiagnosis and ensure prompt, appropriate therapy in aggressive hematologic malignancies.

## Conclusions

DLBCL can present atypically as a rapidly enlarging soft tissue mass, mimicking infection, even without systemic symptoms. For soft tissue lesions unresponsive to standard treatments, malignancy must be strongly considered in the differential diagnosis. Timely imaging and biopsy are essential for accurate diagnosis and prompt initiation of therapy. Additionally, early detection can significantly influence treatment decisions, which is especially important for younger and fitter patients who may be candidates for more aggressive alternative therapies. This report underscores the critical importance of clinical vigilance, multidisciplinary collaboration, and maintaining a low threshold for re-evaluation in atypical or persistent soft tissue lesions, particularly in elderly patients.
